# Genome-Scale Metabolic Models Guided Improvement of Fermented Milk Quality and Flavor by *Lacticaseibacillus paracasei* subsp. *paracasei* 63

**DOI:** 10.3390/foods15111863

**Published:** 2026-05-25

**Authors:** Wenjing Dai, Huandong Yang, Yan Chen, Yi Zou, Zijian Lin, Zihan Fang, Yipeng Tang, Lanyu Qin, Rongjie Zhou, Huafang Xu, Ruixia Gu, Yunchao Wa

**Affiliations:** 1 The School of Food Science and Engineering, Yangzhou University, Yangzhou 225000, China; mx120231310@stu.yzu.edu.cn (W.D.); mz120242238@stu.yzu.edu.cn (Y.C.); 233601333@stu.yzu.edu.cn (Y.Z.); 233601310@stu.yzu.edu.cn (Z.L.); 233601104@stu.yzu.edu.cn (Z.F.); 233601117@stu.yzu.edu.cn (Y.T.); mz120242275@stu.yzu.edu.cn (L.Q.); mz120252263@stu.yzu.edu.cn (R.Z.); guruixia1963@163.com (R.G.); 2 Key Laboratory of Probiotics and Dairy Processing in Provincial Universities, Yangzhou University, Yangzhou 225000, China; 3 Jiangsu Weigang Dairy Research Institute Co., Ltd., Nanjing 210000, China; 807509@wgdairy.com.cn (H.Y.); xuhf@wgdairy.com.cn (H.X.)

**Keywords:** fermented milk, metabolomics, genome-scale metabolic models, microbial metabolic interactions

## Abstract

The quality and flavor of probiotic fermented milk are highly dependent on the strain composition of the starter culture and their metabolic interactions. Although constructing a multi-strain system is an effective strategy for enhancing product quality, traditional formulation methods rely heavily on empirical approaches and lack mechanistic guidance. Herein, this study utilized genome-scale metabolic models (GEMs) to rationally design a multi-strain co-fermentation system. The results demonstrated that the GEM-predicted optimal system, comprising *Lacticaseibacillus paracasei* subsp. *paracasei* 63 (*L. paracasei* subsp. *paracasei* 63) and *Lactococcus cremoris* 290 (*Lc. cremoris* 290), significantly reduced the curd time by approximately 44.0% and 71.0% compared to the *L. paracasei* subsp. *paracasei* 63 and *Lc. cremoris* 290 monocultures, respectively. Furthermore, the co-fermented milk exhibited a 4.3-fold increase in apparent viscosity relative to the 290 single-strain group and achieved a significantly higher diacetyl concentration (1.98 ± 0.09 mg/L), representing a 2.8-fold enhancement. Volatile flavor profiling and untargeted metabolomics provided suggestive evidence supporting the GEM-predicted cross-feeding mechanisms, particularly within the arginine and pyruvate metabolic pathways. This study offers a solid theoretical foundation and practical guidance for the rational design of synthetic microbial communities to develop high-quality fermented dairy products with optimized flavor and functional properties.

## 1. Introduction

The quality of probiotic fermented milk is primarily dictated by the fermentation performance and inherent probiotic properties of the starter cultures [1,2]. Nevertheless, many lactic acid bacteria (LAB) exhibit poor adaptability to the milk matrix, resulting in slow growth, weak acidification, and inadequate synthesis of flavor compounds. These metabolic deficiencies directly compromise the sensory quality and consumer acceptance of the final products [3,4]. Consequently, overcoming these limitations to comprehensively enhance the quality of functional fermented milk has become a critical bottleneck in dairy fermentation engineering. Establishing multi-strain co-fermentation systems to foster metabolic interactions is a proven strategy for improving overall product quality [5,6,7]. From the classic symbiotic pairing of *Streptococcus thermophilus* and *Lactobacillus delbrueckii* subsp. *bulgaricus* to novel starter cultures based on synthetic microbial communities (SynComs), extensive research demonstrates that interspecies metabolic cross-feeding can significantly accelerate acidification, enrich characteristic flavor profiles, and elevate final product quality [8,9,10]. Despite these well-documented benefits, the rational selection and pairing of strains to achieve optimal metabolic synergy remains a significant technical challenge due to the high metabolic specificity of individual LAB strains.

Genome-scale metabolic models (GEMs) are computational frameworks based on whole-genome sequences. They systematically reconstruct microbial biochemical reaction networks and predict metabolic phenotypes [11,12,13]. These models are widely applied to the rational design and optimization of SynComs. For instance, previous studies have constructed GEMs for complex fermentation communities to reveal the central roles of specific strains in supplying nutrients such as amino acids and carbohydrates while maintaining community stability [14,15]. Other research has utilized GEMs for genomic comparison and co-culture simulation of various fermentation strains to identify highly efficient metabolic genes under specific environmental stresses. Currently, GEMs are predominantly applied to large-scale simulations of complex microbial communities. Introducing these models into dairy fermentation starter systems consisting of two to three strains enables an in-depth understanding of interspecies metabolic interaction networks. This approach drives the transition of starter development from empirical trial and error screening to mechanism-driven rational customization.

To address the aforementioned technical challenges, we hypothesized that computational modeling of microbial metabolic interactions could guide the rational design of a superior fermented milk starter system. Consequently, this study employed genome-scale metabolic models (GEMs) to develop a novel co-fermentation system integrating enhanced fermentation performance with inherent probiotic properties. Two distinct lactic acid bacteria strains exhibiting different phenotypes in the milk matrix were selected. We applied GEMs to predict potential metabolic interactions and metabolite exchange networks between these strains and the probiotic *Lacticaseibacillus paracasei* subsp. *paracasei* 63 (*L. paracasei* subsp. *paracasei* 63). These predictions provided a theoretical framework for developing a multi-strain co-fermentation starter culture. An “optimal” synergistic system was then identified based on a multi-criteria objective. This required maximizing computational metabolic complementarity, accelerating fermentation kinetics, and significantly enriching key aroma compounds. Subsequently, we compared the physicochemical properties and flavor profiles of single-strain and multi-strain co-fermented milk. By integrating model predictions with multidimensional untargeted metabolomics, we explored the metabolic response characteristics underlying microbial synergy. Furthermore, we evaluated the correlation between these core metabolic networks and enhanced fermentation performance. These findings serve as a solid theoretical reference for optimizing SynComs in fermented milk, ultimately offering important guiding value for advancing high-quality starter cultures.

## 2. Materials and Methods

### 2.1. Bacterial Strains, Growth Media, and Incubation Conditions

*L. paracasei* subsp. *paracasei* 63, *Lactococcus lactis* subsp. *lactis* 26 (*Lc. lactis* subsp. *lactis* 26), and *Lactococcus cremoris* 290 (*Lc. cremoris* 290, formerly classified as *Lactococcus lactis* subsp. *cremoris* [16]) were used in this study. Both *L. paracasei* subsp. *paracasei* 63 and *Lc. lactis* subsp. *lactis* 26 have be *L. paracasei* subsp. *paracasei* 63en deposited in the China General Microbiological Culture Collection Center (CGMCC) under the accession numbers CGMCC No. 31768 and CGMCC No. 26090, respectively. *Lc. cremoris* 290 is preserved at the Jiangsu Key Laboratory of Probiotics and Dairy Deep Processing. Regarding their origins, *L. paracasei* subsp. *paracasei* 63 was isolated from traditional ghee in the Tibet Autonomous Region, China; *Lc. lactis* subsp. *lactis* 26 was isolated from milk curd in the Inner Mongolia Autonomous Region, China; and *Lc. cremoris* 290 was obtained from traditional fermented milk in the Inner Mongolia Autonomous Region, China. The tested strains were subcultured for three consecutive generations at 30 °C in their respective MRS or M17 media. Subsequently, the cultures were subjected to centrifugation, washing, and resuspension. Viable plate counting was then performed to prepare standardized bacterial suspensions for subsequent experiments.

### 2.2. Production and Storage of Fermented Milk

Commercially available whole UHT bovine milk (protein: 3.2 g/100 mL; fat: 3.8 g/100 mL; carbohydrate: 4.8 g/100 mL) was used as the base matrix. This milk was supplemented with 7% (*w*/*w*) sucrose to simulate commercial sweetened formulations and improve the texture of the milk matrix, resulting in an estimated total dry matter content of approximately 19.5%. The supplemented milk was then heated at 95 °C for 5 min and cooled to ambient temperature for the subsequent fermentation trials. The experimental setup comprised multi-strain co-fermentation groups utilizing a 1:1 inoculation ratio of the paired strains, alongside single-strain fermentation groups for each tested strain. Detailed grouping configurations are outlined in Table 1. All samples were inoculated to achieve a final viable count of 2 × 10^7^ CFU/mL. For each experimental trial, 100 mL of the inoculated milk was dispensed into a 200 mL food-grade polypropylene (PP) sealed jar, maintaining a precise liquid-to-headspace volume ratio of 1:1. The inoculated milk was fermented at 30 °C, with the pH monitored every 2 h. The fermentation endpoint (EF) was determined upon visual confirmation of complete coagulation, which corresponded to a pH of approximately 4.5 ± 0.2 (notably, fermentation was terminated at 48 h for slow-acidifying groups, such as the single-strain 290 group). Following fermentation, all samples underwent a 24-h cold storage period at 4 °C. The completion of this period was designated as the ripening endpoint (ER). All independent fermentation trials were performed in three biological replicates. Furthermore, for each biological replicate, parameter monitoring and subsequent sample analyses were conducted using three technical replicates.

### 2.3. Strain Sequence Analysis

All tested strains were cultured to the logarithmic growth phase in their respective liquid media. Bacterial cell pellets were then harvested from the liquid culture broth via centrifugation at 14,000× *g* for 5 min at ambient temperature using an H1750R high-speed refrigerated centrifuge (Hunan Xiangyi Laboratory Instrument Development Co., Ltd., Changsha, China). Total genomic DNA was extracted from each pellet utilizing a commercial bacterial genomic DNA extraction kit in strict accordance with the manufacturer’s protocol. The integrity, purity, and concentration of the extracted DNA were assessed using 0.6% agarose gel electrophoresis, a NanoDrop 2000 spectrophotometer (Thermo Fisher Scientific, Waltham, MA, USA), and a Quant^TM^ fluorometer (Promega, Madison, WI, USA) coupled with a PicoGreen fluorescence assay, respectively. Only DNA samples meeting the rigorous quality requirements were selected for downstream sequencing.

Whole-genome sequencing of the tested strains utilized a combination of next-generation sequencing (NGS) and third-generation sequencing (TGS). These services were provided by Shanghai Majorbio Bio-pharm Technology Co., Ltd (Majorbio, Shanghai, China). The NGS was executed on an Illumina NovaSeq 6000 platform (Illumina Inc, San Diego, CA, USA) utilizing a paired-end 150 bp read mode at a minimum sequencing depth of 100×. Concurrently, the TGS was conducted on a PacBio Sequel IIe platform (Pacific Biosciences, Menlo Park, CA, USA) at a minimum depth of 50× following the successful construction of SMRTbell libraries.

After quality control and filtering of the raw reads, a hybrid de novo genome assembly was performed utilizing Unicycler v0.4.8 to integrate the quality-controlled NGS and TGS data. To generate the complete genomic sequence of each strain, SOAPdenovo2 v2.04 assisted in assembly optimization, GapCloser v1.12 filled gaps within the assembled scaffolds, and Pilon v1.22 executed single base error corrections. The quality of these assembled genomes was then evaluated using CheckM v1.2.2 based on core metrics including genome completeness, contamination rate, and the N50 value. Finally, high-quality genome sequences satisfying all criteria were selected for subsequent analysis and stored in FASTA format.

### 2.4. Generation and Simulation of GEMs

Metabolic modeling was performed utilizing the prokaryotic GEM reconstruction and simulation module within the metaGEM workflow [17,18]. Considering the relatively short fermentation period of the system, all metabolic simulations were conducted in a standardized “milk medium” matrix [17]. Initially, Prodigal v2.6.3 [19] generated protein sequence files annotated with open reading frames (ORFs) from the corresponding genomic DNA FASTA files of each tested strain. Subsequently, CarveMe v1.4.1 [20] constructed the GEMs for *L. paracasei* subsp. *paracasei* 63, *Lc. lactis* subsp. *lactis* 26, and *Lc. cremoris* 290. The quality of these constructed models was then evaluated using MEMOTE v0.13.0 [21]. Furthermore, reframed v1.2.1 and cobrapy v0.20.0 [22,23] executed the single-strain simulations under the aforementioned milk medium conditions. Finally, to investigate the metabolic interactions within the microbial community in the milk medium matrix, SMETANA v1.2.0 [24] facilitated the community-level GEM simulations.

### 2.5. Analysis of pH and Titratable Acidity (TA) in Fermented Milk

The pH values of all fermented milk samples were measured at both the EF and the ER using a FiveEasy Plus FE28 pH meter (Mettler-Toledo, Greifensee, Switzerland)). Concurrently, the titratable acidity (TA, expressed in degrees Thorner, °T) of the same samples was quantified at these two stages via titration with a 0.1 mol/L standardized sodium hydroxide (NaOH) solution, utilizing 0.5% (*w*/*v*) phenolphthalein as the indicator [25]. The TA was calculated using the following formula:

(1) $$\mathrm{TA}({}^{\circ}T)\;=\;\frac{c\times V\times 100}{m\times 0.1},$$

where *c* is the exact concentration of the standardized NaOH solution (mol/L), *V* is the volume of the NaOH solution consumed during the titration (mL), *m* is the mass of the fermented milk sample (g), and 0.1 is the reference concentration of NaOH defining the Thorner degree (mol/L).

### 2.6. Determination of the Viable Count of Microorganisms in Fermented Milk

The viable counts of lactic acid bacteria were determined at both the EF and the ER using serial dilution and plate spreading methods on de Man, Rogosa, and Sharpe (MRS) agar [26]. To account for strain-specific differences in optimal growth temperatures, a differential counting approach was employed to evaluate the viable lactic acid cocci and bacilli within the fermented milk samples [27]. Specifically, total viable counts (comprising both *L. paracasei* subsp. *paracasei* 63 and *Lactococcus* strains) were determined following incubation on MRS agar at 30 °C for 48 h. Simultaneously, the *L. paracasei* subsp. *paracasei* 63 bacilli were selectively enumerated by incubating the MRS plates at 45 °C for 48 h, a condition that completely inhibits the mesophilic Lactococcus strains. The viable count of the *Lactococcus* cocci was subsequently calculated by subtracting the *L. paracasei* subsp. *paracasei* 63 count from the total count at 30 °C.

### 2.7. Apparent Viscosity of Fermented Milk

The apparent viscosity of all fermented milk samples was measured exclusively at the ER at a fixed shear rate of 0.1 s^−1^ through steady-state shear rate sweep tests [28]. These measurements were performed using a Kinexus Pro rotational rheometer (Malvern Panalytical Ltd., Malvern, UK) at a constant temperature of 25 °C.

### 2.8. Determination of Diacetyl Content in Fermented Milk

The diacetyl content of all fermented milk samples was quantified exclusively at the ER using the *o*-phenylenediamine (OPDA) colorimetric method [29,30]. This assay relies on the specific reaction between OPDA and diacetyl to yield 2,3-dimethylquinoxaline, a compound exhibiting a maximum absorption wavelength at 335 nm. To initiate the extraction, proteins within the samples were precipitated utilizing trichloroacetic acid, followed by centrifugation to collect the supernatant. OPDA was then introduced to the supernatant, and the resulting mixture was incubated in the dark for 30 min. Following the addition of hydrochloric acid to terminate the reaction, the optical density of the mixture was measured at 335 nm (OD_335_).

### 2.9. Sensory Evaluation

The sensory evaluation protocol was conducted in accordance with the Declaration of Helsinki and the ethical guidelines of Yangzhou University. Informed consent was obtained from all participants prior to the sensory evaluation. The sensory panel comprised 12 trained individuals (aged 20–30 years) with an equal gender distribution of six males and six females. Prior to the formal assessment, all panelists completed a minimum of 20 h of systematic training with standard sensory reference materials for fermented dairy products. This rigorous preparation ensured the consistency and repeatability of the scoring results. Following the completion of the cold storage period at 4 °C, all fermented milk samples were immediately retrieved and evaluated exclusively at the ER. The panelists utilized a 9-point structured hedonic scale to assess the flavor and texture attributes of the samples [31], with a score of 9 representing excellent quality and a score of 1 indicating poor quality. Unsalted crackers and room temperature water were provided for palate cleansing between sample evaluations.

### 2.10. Analysis of Volatile Compounds in Fermented Milk

Headspace solid-phase microextraction coupled with gas chromatography-mass spectrometry (HS-SPME-GC-MS) was employed to analyze the volatile and semi-volatile flavor compounds in all fermented milk samples exclusively at the ER. Prior to extraction, a 50/30 μm CAR/DVB/PDMS extraction fiber was conditioned at 250 °C for 15 min. A 5 g aliquot of each sample was transferred into a 20 mL headspace vial, followed by the addition of 5 μL of the internal standard *o*-dichlorobenzene and 1.0 g of sodium chloride (NaCl). The vial was immediately sealed with a polytetrafluoroethylene (PTFE)/silica gel septum and an aluminum crimp cap. Subsequently, the samples were equilibrated at 45 °C for 20 min and subjected to extraction at the same equilibrium temperature for 40 min.

For chromatographic separation, a DB-WAX capillary column (30 m × 0.25 mm internal diameter, 0.25 μm film thickness) was utilized. The column oven temperature program commenced at 40 °C for 3 min, ramped to 140 °C at a rate of 5 °C/min with a 1 min hold, and further increased to 250 °C at a rate of 10 °C/min with a final 5 min hold. The inlet temperature was maintained at 250 °C. High-purity helium served as the carrier gas at a constant flow rate of 1.0 mL/min under a splitless injection mode.

Mass spectrometry was conducted utilizing an electron impact (EI) ionization mode at an energy of 70 eV. The ion source temperature was set to 220 °C. Mass spectra were acquired over a scanning range of 30.00 to 500.00 *m*/*z*, utilizing an emission current of 100 μA and a detector voltage of 1.4 kV.

For qualitative and quantitative analysis, the mass spectrum of each detected compound was identified through automated matching against the NIST 2.2 mass spectral library. The concentrations of all target compounds were subsequently quantified utilizing the internal standard method.

### 2.11. Analysis of Untargeted Metabolomics in Fermented Milk

For all fermented milk samples, untargeted metabolomic analysis was conducted exclusively at the ER. The analysis was conducted by Shanghai Majorbio Bio-pharm Technology Co., Ltd. Chromatographic separation and mass spectrometric detection were performed using an ultra-high-performance liquid chromatography (UHPLC) system coupled with an Exactive HF-X high-resolution mass spectrometer (Thermo Fisher Scientific, USA).

For chromatographic separation, an HSS T3 column (100 mm × 2.1 mm internal diameter, 1.8 μm particle size; Waters Corporation, Milford, MA, USA) was utilized and maintained at 40 °C. Mobile phase A consisted of a 5% aqueous acetonitrile solution containing 0.1% formic acid by volume. Mobile phase B comprised a mixture of 47.5% acetonitrile, 47.5% isopropanol, and 5% water, supplemented with 0.1% formic acid by volume. A gradient elution program with variable flow rates was established as follows: from 0 to 3.5 min, mobile phase B was linearly increased from 0% to 24.5% at a flow rate of 0.40 mL/min; from 3.5 to 5 min, it was increased to 65% at 0.40 mL/min; from 5 to 5.5 min, it reached 100% at 0.40 mL/min; from 5.5 to 7.4 min, it was maintained at 100% while the flow rate increased to 0.6 mL/min; from 7.4 to 7.6 min, it was decreased to 51.5% at 0.6 mL/min; from 7.6 to 7.8 min, it dropped to 0% at 0.5 mL/min; from 7.8 to 9 min, it remained at 0% with the flow rate adjusted to 0.40 mL/min; and finally, from 9 to 10 min, it was maintained at 0% at a flow rate of 0.40 mL/min.

Mass spectrometry signal acquisition employed both positive and negative electrospray ionization (ESI) modes. Key parameters included a capillary spray voltage of 3.5 kV for the positive ion mode and −3.5 kV for the negative ion mode. The ion transfer tube temperature was maintained at 325 °C, and the stepped normalized collision energy (NCE) for MS2 fragmentation was configured to 20, 40, and 60 V. First-stage mass spectrometry (MS1) operated at a resolution of 60,000 across a scanning range of 70 to 1050 *m*/*z*, while second-stage mass spectrometry (MS2) operated at a resolution of 7500. All data were collected utilizing a data-dependent acquisition (DDA) mode [32].

For data processing and bioinformatics analysis, the MajorBio Cloud platform (cloud.majorbio.com) was utilized. Raw mass spectrometry data from both ionization modes were imported into Progenesis QI v2.0 software (Waters Corporation, USA) for preprocessing, and metabolite identification was achieved by matching the spectral information against the Human Metabolome Database (HMDB, http://www.hmdb.ca/, accessed on 8 April 2026). Partial least squares discriminant analysis (PLS-DA) was executed utilizing the ropls package (v1.6.2) within the R software environment. Differentially accumulated metabolites (DAMs) were identified based on dual-screening criteria: a variable importance in projection (VIP) score greater than 1 from the PLS-DA model and a false discovery rate (FDR) corrected *p*-value of less than 0.05. Metabolic pathway annotation and mapping of these DAMs relied on the Kyoto Encyclopedia of Genes and Genomes (KEGG) PATHWAY Database (https://www.kegg.jp/kegg/pathway.html (accessed on 8 April 2026)). Finally, pathway enrichment analysis was conducted using the scipy.stats package in Python (v1.10.0), identifying the biological pathways most significantly associated with the experimental treatments via Fisher’s exact test.

### 2.12. Statistical Analysis

All routine experiments and parameter measurements were performed in three independent biological replicates. To ensure the robustness of the high-dimensional data, the untargeted metabolomics analyses were conducted with six independent biological replicates. All results are expressed as the mean ± standard deviation (SD). Statistical evaluations were executed utilizing a one-way analysis of variance (ANOVA) within IBM SPSS Statistics v23 (IBM Corp., Armonk, NY, USA). Subsequent multiple comparisons among groups were assessed via the Waller–Duncan post hoc test, where a *p*-value of less than 0.05 indicated statistical significance. Data visualization was accomplished utilizing Origin 2024 (OriginLab Corp., Northampton, MA, USA).

## 3. Results

### 3.1. Genome Sequencing and Assembly Quality Assessment

The genomic DNA extracted from all tested strains fulfilled the rigorous prerequisites for library construction. Whole-genome sequencing of the three strains generated a total of 3.22 Gb of clean NGS data and 1.04 Gb of clean TGS data. The Q30 scores across all sequencing datasets exceeded 97%, achieving a genomic coverage greater than 99.99%. Following a hybrid de novo assembly, CheckM and BUSCO evaluated the quality of the assembled genomes. These assessments confirmed a genome completeness of at least 99.46% via CheckM, a complete single-copy ortholog proportion of 97.6% or higher via BUSCO, and a contamination rate strictly below 0.95%. Ultimately, these high-quality genome assemblies thoroughly satisfy the criteria for the accurate reconstruction of GEMs in subsequent analyses. Detailed assembly metrics are available in Table S1 of the Supplementary Materials.

### 3.2. Prediction of Strain Interactions Based on GEMs

**Figure 1:**
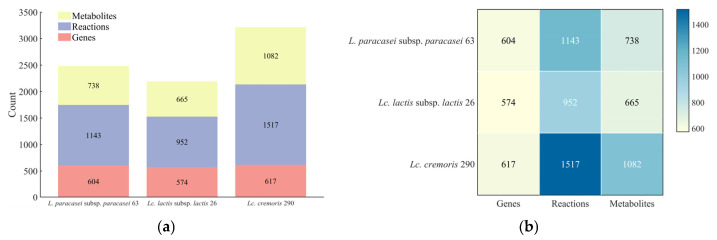
Genome-scale metabolic models of *L. paracasei* subsp. *paracasei* 63, *Lc. lactis* subsp. *lactis* 26, and *Lc. cremoris* 290. (**a**) Model component analysis; (**b**) Distribution patterns of the models, where dark regions represent reaction numbers, medium-intensity regions indicate metabolites, and light regions show gene counts.

GEMs for *L. paracasei* subsp. *paracasei* 63, *Lc. lactis* subsp. *lactis* 26, and *Lc. cremoris* 290 were constructed in this study. An analysis of the metabolic network components revealed significant interspecies differences among the three strains (Figure 1a). Furthermore, a heatmap analysis quantified the distribution characteristics of these components (Figure 1b). The results demonstrated that *Lc. cremoris* 290 possessed the most complex metabolic network, comprising 617 genes, 1517 reactions, and 1082 metabolites. In contrast, *Lc. lactis* subsp. *lactis* 26 exhibited a comparatively simpler network containing 574 genes, 952 reactions, and 665 metabolites.

**Figure 2:**
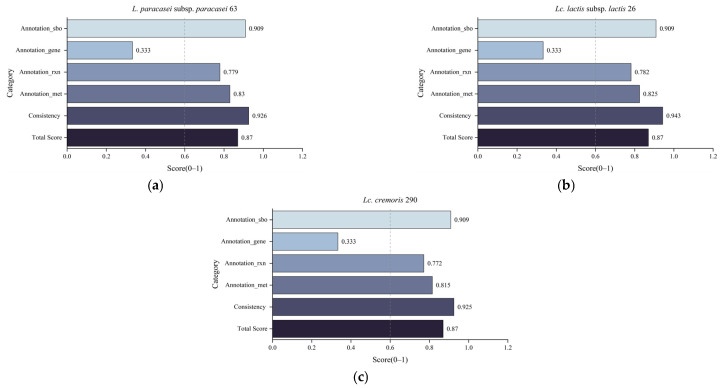
Model quality assessment scores for three bacterial strains’ genome-scale metabolic models. The quality of genome-scale metabolic models for three bacterial strains was evaluated using multiple metrics. (**a**) Model quality assessment scores for *L. paracasei* subsp. *paracasei* 63; (**b**) Model quality assessment scores for *Lc. lactis* subsp. *lactis* 26; (**c**) Model quality assessment scores for *Lc. cremoris* 290. The vertical dashed line in each panel represents a quality reference threshold of 0.6.

The MEMOTE suite assessed the quality of the constructed GEMs and confirmed their high reliability for all three tested strains. Detailed evaluation results are presented in Figure 2. Specifically, the mass balance consistency score for each model exceeded 0.92, the overall quality score reached an average of 0.87, and the annotation scores for all strains surpassed 0.90. These consistently high scores across all core evaluation metrics provided a reliable computational foundation for the subsequent simulation of interspecies metabolic interactions within the milk medium matrix.

**Figure 3:**
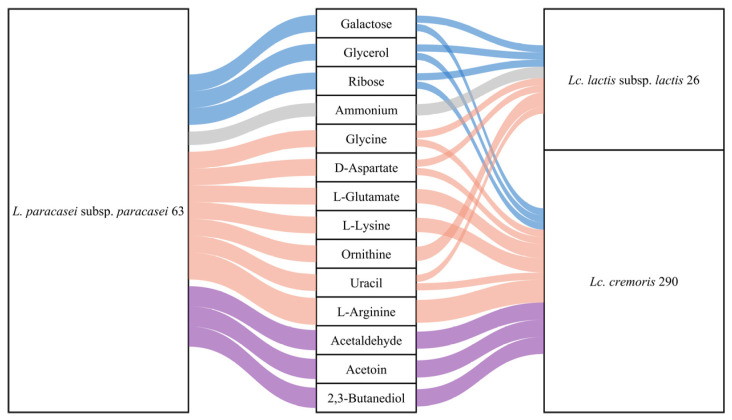
Predicted metabolic network interactions between two lactic acid bacteria in different co-fermentation groups using GEMs. The width of the arrows indicates the relative magnitude of metabolic flux, and different colors represent different metabolic pathways.

To identify the optimal combination with the highest fermentation potential, the SMETANA algorithm predicted the metabolic interactions between all feasible two-strain pairings within a simulated milk medium (Figure 3). In silico predictions indicated that both *Lc. lactis* subsp. *lactis* 26 and *Lc. cremoris* 290 could supply *L. paracasei* subsp. *paracasei* 63 with multiple B vitamins, namely folic acid, pantothenic acid, and niacin, alongside essential amino acids like L-leucine and L-phenylalanine. This cross-feeding established the nutritional foundation for the stable growth of *L. paracasei* subsp. *paracasei* 63 within the co-culture system. In comparison, the 63 + 290 co-fermentation group exhibited a more complex and extensive metabolic cross-feeding network. Specifically, the models predicted that *L. paracasei* subsp. *paracasei* 63 exclusively transferred L-arginine to *Lc. cremoris* 290 within this system. This transfer indicates a potential synergistic effect between the two strains in regulating acid stress tolerance. Furthermore, although *L. paracasei* subsp. *paracasei* 63 was predicted to transfer L-aspartic acid and L-asparagine to its partner strains in both the 63 + 26 co-fermentation group and the 63 + 290 co-fermentation group, the exchange intensity of these two amino acids was notably higher in the latter. This intensified exchange reflects a deeper metabolic coupling regarding nitrogen source metabolism and amino acid cycling. Notably, the in silico cross-feeding of key flavor precursors, including acetaldehyde, acetone, and (R,R)-2,3-butanediol, was exclusively detected within the metabolic interaction network of the 63 + 290 co-fermentation group. This distinct feature suggests potential synergistic effects between the two strains within the pyruvate metabolic pathway, thereby possessing greater application potential for enhancing the volatile flavor profile of the resulting fermented milk.

### 3.3. The Effect of Mixed Strains on the Quality of Fermented Milk

Following the co-inoculation of *L. paracasei* subsp. *paracasei* 63 with *Lc. lactis* subsp. *lactis* 26, the curd time decreased to a duration comparable to that of the *Lc. lactis* subsp. *lactis* 26 single-strain fermentation (Table 2). In contrast, co-culturing *L. paracasei* subsp. *paracasei* 63 with *Lc. cremoris* 290 substantially accelerated the fermentation process, reducing the curd time by approximately 44% and 71% relative to the *L. paracasei* subsp. *paracasei* 63 and *Lc. cremoris* 290 single-strain fermentation groups, respectively. At the EF, the 63 + 290 co-fermentation group exhibited the highest total viable cell count. Notably, the viable count of *Lc. cremoris* 290 within this mixed system was significantly greater than that observed in its monoculture counterpart (*p* < 0.05). Furthermore, throughout the 24-h cold storage period at 4 °C, the 63 + 290 co-fermentation group maintained remarkable stability, exhibiting no significant alterations in either viable cell counts or post-acidification levels.

**Figure 4:**
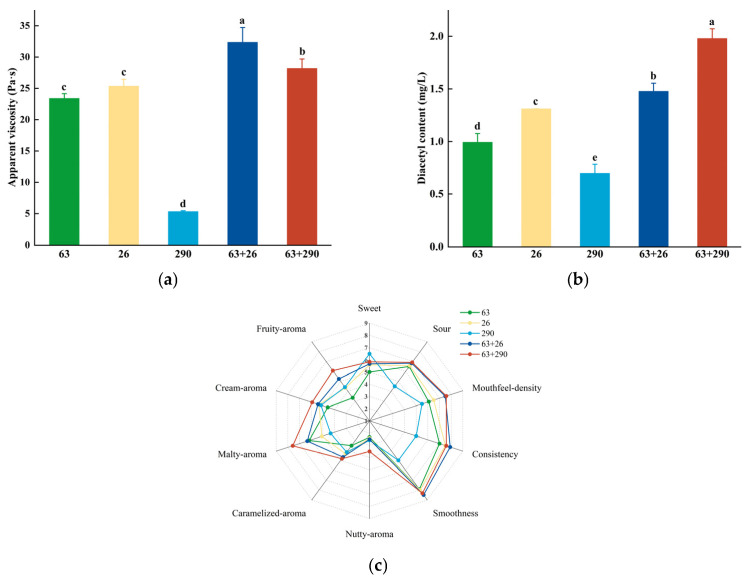
Apparent viscosity, diacetyl content and sensory attributes of fermented milk samples fermented with single-strains and co-cultures. (**a**) Apparent viscosity; (**b**) Diacetyl content; (**c**) Sensory radar plot. Different lowercase letters above the bars indicate significant differences between groups (*p* < 0.05).

Following coagulation, the fermented milk samples underwent a 24-h cold storage period at 4 °C prior to the evaluation of their apparent viscosity, diacetyl content, and sensory attributes at the ER. The results demonstrated that the apparent viscosity of the co-fermented milk samples increased significantly compared to their monoculture counterparts (Figure 4a, *p* < 0.05). Specifically, the 63 + 26 co-fermentation group exhibited the highest apparent viscosity among all groups, while the viscosity of the 63 + 290 co-fermentation group was approximately 4.3-fold higher than that of the 290 single-strain group. Furthermore, significant variations in diacetyl accumulation were observed across the different experimental groups (Figure 4b, *p* < 0.05). Co-fermentation universally enhanced diacetyl production, with the 63 + 290 co-fermentation group achieving the highest concentration (1.98 ± 0.09 mg/L), representing an approximate 2.8-fold increase relative to the 290 single-strain group.

As illustrated in the radar chart (Figure 4c), the sensory evaluation encompassed 10 attributes across the various fermented milk groups: fruity, buttery, malty, nutty, and caramel aromas, alongside sweetness, acidity, flavor intensity, body, and mouth smoothness. The findings indicated that the overall sensory quality of the co-fermented milk samples generally surpassed that of the single-strain fermentation groups. Specifically, the 63 + 290 co-fermentation group excelled across multiple sensory dimensions, achieving significantly higher scores for the fruity, buttery, malty, and nutty aromas compared to all other samples (*p* < 0.05). In contrast, while the 63 + 26 co-fermentation group enhanced the textural properties of the fermented milk, it exerted no significant promoting effect on the overall aroma profile of the product.

### 3.4. Differences in Volatile Compound Profiles Between Single-Strain Fermented Milk and Co-Cultured Fermented Milk

**Figure 5:**
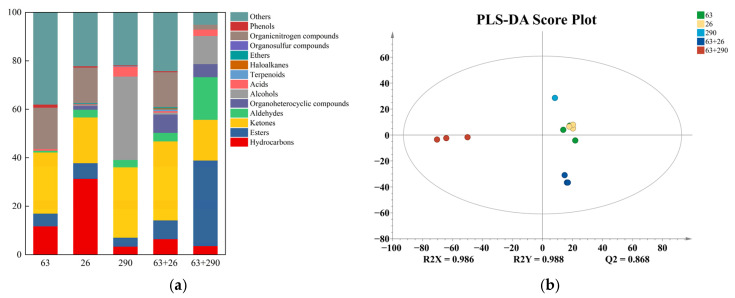
Classification and relative abundance of volatile compounds and partial least squares discriminant analysis (PLS-DA) score plot in fermented milk samples. (**a**) Classification and relative abundance of volatile compounds identified in fermented milk samples; (**b**) PLS-DA score plot derived from volatile compound data.

HS-SPME-GC-MS analysis identified a total of 85 volatile compounds across all fermented milk samples. These compounds comprised 18 hydrocarbons, 17 esters, 7 ketones, 5 aldehydes, 5 carboxylic acids, 5 alcohols, 4 heterocyclic organic compounds, 3 terpenoids, and 21 miscellaneous compounds. The detailed identification and relative quantification of all 85 volatile compounds across the different experimental groups are provided in Supplementary Table S2. Figure 5a illustrates the relative abundance distribution of these volatile compounds across the various experimental groups. Following the co-inoculation of *L. paracasei* subsp. *paracasei* 63 with the Lactococcus strains, the relative contents of ketones and heterocyclic compounds within the 63 + 26 co-fermentation group increased significantly compared to their corresponding single-strain counterparts (*p* < 0.05). Conversely, the 63 + 290 co-fermentation group exhibited a significant elevation in the relative contents of esters and aldehydes (*p* < 0.05).

A PLS-DA score plot of the volatile metabolite profiles revealed tight intra-group aggregation alongside significant inter-group separation (Figure 5b). The *L. paracasei* subsp. *paracasei* 63 and *Lc. lactis* subsp. *lactis* 26 monoculture groups clustered tightly together in spatial proximity, indicating highly similar volatile flavor profiles. In contrast, the *Lc. cremoris* 290 monoculture was spatially distinct from these two groups, presenting a relatively unique flavor characteristic. Upon co-inoculating *L. paracasei* subsp. *paracasei* 63 with *Lc. lactis* subsp. *lactis* 26 and *Lc. cremoris* 290, respectively, both resulting co-fermentation systems developed unique flavor profiles clearly distinguishable from those of the single-strain fermentations. The PLS-DA model demonstrated excellent fitting and predictive performance, yielding a dependent variable fitting parameter (R^2^Y) of 0.988, an independent variable fitting parameter (R^2^X) of 0.986, and a model predictive capability index (Q^2^) of 0.868. Furthermore, a 200-iteration permutation test supported the absence of model overfitting (Figure S1).

**Figure 6:**
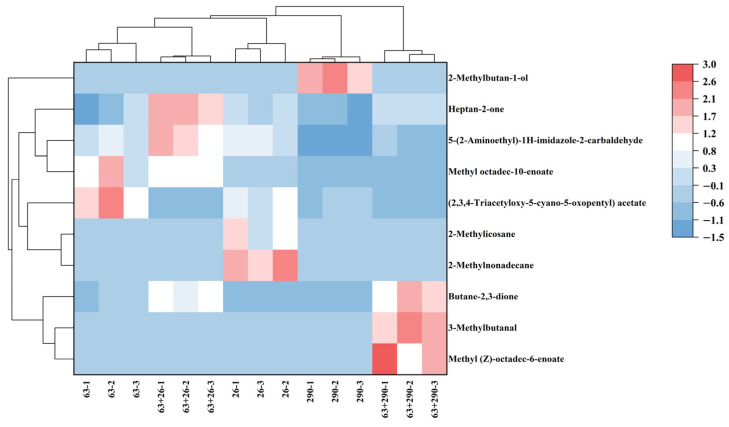
Heatmap of the top 10 volatile compounds with the highest variable importance in projection (VIP) scores. Heatmap visualization showing the row-normalized relative abundances of volatile compounds in different fermented milk samples. The color gradient from blue (low abundance) to red (high abundance) represents the normalized relative content of each volatile compound.

Differentially accumulated volatile metabolites were screened utilizing a VIP threshold greater than 1, based on the aforementioned PLS-DA model. Figure 6 illustrates the top 10 volatile compounds ranked in descending order by their VIP scores. Distinct characteristic metabolite profiles emerged across the various fermentation systems. These variations indicated that co-inoculation with *Lactococcus* strains significantly altered the volatile flavor metabolome of the *L. paracasei* subsp. *paracasei* 63 fermented milk (*p* < 0.05). Notably, diacetyl (2,3-butanedione) and 3-methylbutyraldehyde exhibited high relative abundances within the 63 + 290 co-fermentation group. The compound diacetyl imparts a rich creamy and buttery aroma typical of fermented milk, whereas 3-methylbutyraldehyde contributes mild malty and fruity notes. Together, these compounds synergistically shape the unique flavor profile of this specific microbial combination. In contrast, the 63 + 26 co-fermentation group presented a higher relative abundance of 2-heptanone, a metabolite that confers characteristic fruity and cheesy aromas to the final product.

### 3.5. Differences in Metabolomic Profiles Between Single-Strain Fermented Milk and Co-Cultured Fermented Milk

**Figure 7:**
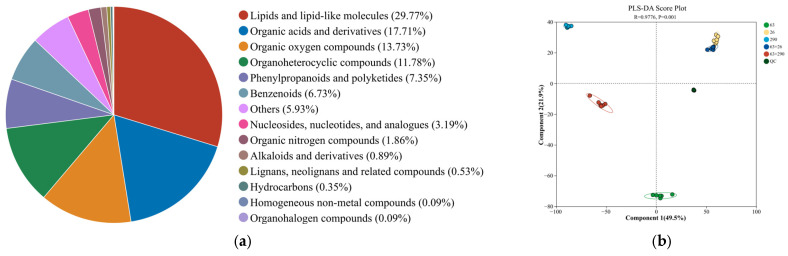
Classification of untargeted metabolites and partial least squares discriminant analysis (PLS-DA) score plot in fermented milk samples. (**a**) Classification of all identified untargeted metabolites in fermented milk samples; (**b**) PLS-DA score plot of untargeted metabolomic profiles from different fermented milk samples.

Untargeted metabolomics analysis identified a total of 1065 metabolites across all fermented milk samples collected at the ER, predominantly comprising lipids, organic acids, and heterocyclic compounds, alongside other minor categories (Figure 7a). A PLS-DA score plot of the untargeted metabolite profiles revealed tight intra-group clustering and significant inter-group separation. This distinct spatial distribution clearly differentiated the fermented milk samples across the various experimental groups, highlighting profound shifts in their global metabolic profiles (Figure 7b). The tight clustering of quality control (QC) samples within the score plot suggested an excellent stability and analytical reproducibility of the metabolomics workflow. Among the experimental configurations, the *Lc. lactis* subsp. *lactis* 26 monoculture and the 63 + 26 co-fermentation group were spatially adjacent and clustered closely together. Conversely, all other fermentation groups were clearly separated from one another. The PLS-DA model demonstrated excellent fitting and predictive performance, yielding a dependent variable fitting parameter (R^2^Y) of 0.853, an independent variable fitting parameter (R^2^X) of 0.992, and a model predictive capability index (Q^2^) of 0.949. Furthermore, a 200-iteration permutation test supported the absence of model overfitting (Figure S2).

**Figure 8:**
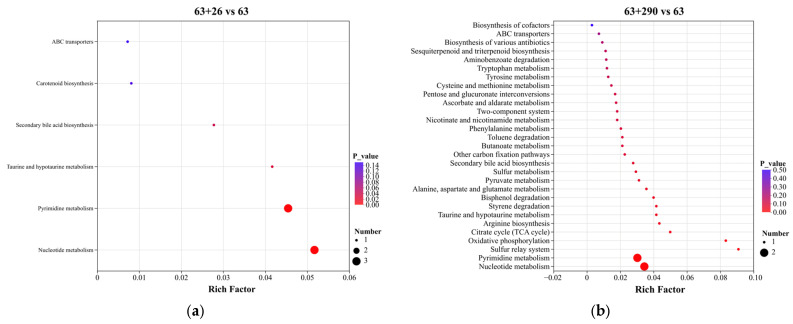
KEGG enrichment results for differentially expressed non-target metabolites. (**a**) KEGG enrichment results for differentially expressed non-target metabolites between the 63 + 26 co-fermentation group and 63 group; (**b**) KEGG enrichment results for differentially expressed non-target metabolites between the 63 + 290 co-fermentation group and 63 group.

Based on the aforementioned PLS-DA model, a total of 224 DAMs were identified relative to the *L. paracasei* subsp. *paracasei* 63 monoculture. This identification utilized rigorous screening criteria: VIP > 1.0, fold change (FC) > 1.5 or FC < 0.667, and FDR < 0.05. Subsequent metabolic pathway enrichment analysis (Figure 8) revealed distinct metabolic preferences among the co-fermentation groups, reflecting their unique metabolic interaction patterns. The 63 + 26 co-fermentation group exhibited significantly fewer perturbed metabolic pathways, which were predominantly concentrated within nucleotide and pyrimidine metabolism (*p* < 0.05). This finding suggests that the co-fermentation of *L. paracasei* subsp. *paracasei* 63 and *Lc. lactis* subsp. *lactis* 26 did not trigger a global reorganization of carbon and nitrogen metabolic fluxes; instead, it induced metabolic interactions primarily limited to maintaining cell growth and basal metabolism.

In contrast, the co-fermentation of *L. paracasei* subsp. *paracasei* 63 and *Lc. cremoris* 290 resulted in the significant enrichment of numerous core metabolic pathways (*p* < 0.05). These encompassed key pathways involved in amino acid degradation and conversion, specifically alanine, aspartate, and glutamate metabolism; arginine biosynthesis; tyrosine metabolism; tryptophan metabolism; as well as cysteine and methionine metabolism. Concurrently, core carbon and energy metabolism pathways, including the tricarboxylic acid (TCA) cycle and oxidative phosphorylation, demonstrated extremely high enrichment significance (*p* < 0.01). The aforementioned amino acids serve as essential precursors for the biosynthesis of characteristic volatile flavor compounds, including higher alcohols, aldehydes, and esters within the fermented milk. Furthermore, the activation of the TCA cycle supplies abundant precursor substrates and energy facilitating the esterification reactions of these flavor compounds.

**Figure 9:**
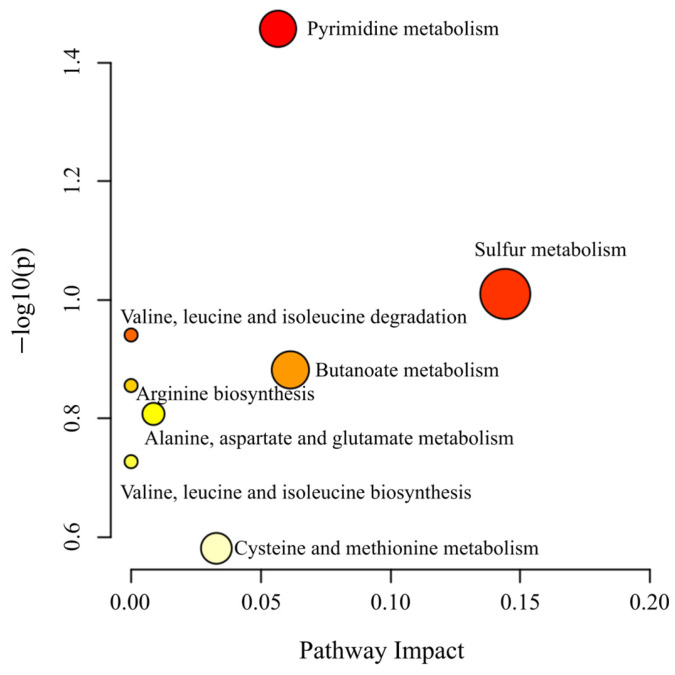
KEGG pathway analysis of differential compounds in fermented milk.

To further elucidate the underlying metabolic interaction mechanisms within the 63 + 290 co-fermentation group, which exhibited optimal flavor enhancement, an integrated analysis of its volatile and non-volatile metabolite profiles was conducted. A total of 80 metabolites demonstrated significant differential abundance relative to the *L. paracasei* subsp. *paracasei* 63 monoculture based on the established screening criteria. These criteria included VIP > 1.0, a FC > 1.5 or FC < 0.667, and FDR < 0.05. Specifically, 14 metabolites were up-accumulated, whereas 66 were down-accumulated (Table S3). Subsequent metabolic pathway enrichment analysis (Figure 9) revealed that these DAMs were predominantly enriched (*p* < 0.05) within several critical pathways. These encompassed pyrimidine metabolism, sulfur metabolism; butyrate metabolism; the degradation and biosynthesis of branched-chain amino acids (BCAAs), including valine, leucine, and isoleucine; arginine biosynthesis; alanine, aspartate, and glutamate metabolism; as well as cysteine and methionine metabolism.

## 4. Discussion

The overall quality of probiotic fermented milk heavily relies on the complex metabolic interaction networks within the microbial community of the starter cultures [33]. However, existing single-strain starters frequently fail to balance excellent probiotic functionality with ideal fermentation performance [34]. Consequently, overcoming the limitations of traditional empirical trial and error screening to rationally design novel multi-strain fermentation systems with precise metabolic synergy has emerged as a key technical challenge in dairy fermentation engineering. This study integrated GEMs with multidimensional experimental validation to evaluate a potential synergistic fermentation system. The findings demonstrated that *Lc. cremoris* 290 served as the optimal synergistic strain among tested for co-fermentation with *L. paracasei* subsp. *paracasei* 63. Their metabolic complementarity and cross-feeding interactions significantly reshaped the physicochemical properties and volatile flavor profile of the fermented milk. Ultimately, these interactions strongly influenced the overall quality and sensory characteristics of the final product.

In this study, GEMs were constructed for *L. paracasei* subsp. *paracasei* 63, *Lc. lactis* subsp. *lactis* 26, and *Lc. cremoris* 290 to simulate community level metabolic interactions within a standard milk medium. The simulation results indicated that both *Lactococcus* strains provided 11 B vitamins and essential amino acids to support the growth of *L. paracasei* subsp. *paracasei* 63. In return, *L. paracasei* subsp. *paracasei* 63 maintained the stability of the community metabolism by consuming environmental oxygen and supplying trace elements [35,36]. Notably, a more profound metabolic coupling emerged between *L. paracasei* subsp. *paracasei* 63 and *Lc. cremoris* 290. Model predictions indicated that *L. paracasei* subsp. *paracasei* 63 directed the cross-feeding of L-arginine, L-aspartic acid, L-asparagine, and L-threonine to *Lc. cremoris* 290 alongside basic amino acid exchange. Arginine metabolism generates ATP via the arginine deiminase (ADI) pathway and regulates intracellular pH. This process significantly enhances the adaptability of the strains under acidic stress conditions [37]. Furthermore, the highly efficient exchange of sulfate between the two strains and the cross-species transfer of key flavor precursors like acetaldehyde, diacetyl, and ethyl acetoacetate suggest potential synergistic interactions within sulfur-containing amino acid synthesis and the pyruvate metabolic pathway [38].

Subsequent validation of the fermentation trials demonstrated that, compared to fermentations utilizing a single strain, the multi-strain co-fermentation system significantly accelerated the fermentation rate and reduced the curd time [39]. Simultaneously, this synergistic approach enhanced both strain viability and the apparent viscosity of the fermented milk. Notably, diacetyl accumulation within the 63 + 290 co-fermentation group exhibited a substantial increase. This empirical observation aligned well with the prior computational model predictions.

The distinctive fruity, malty, and milky aromas of the 63 + 290 co-fermentation group stemmed from the redistribution of carbon and nitrogen metabolism alongside pyruvate flux at the community level. Particularly regarding carbon metabolism, although *L. paracasei* subsp. *paracasei* 63 is inherently facultatively heterofermentative, the hexose-rich environment (milk lactose and 7% sucrose) strongly prioritized the homolactic EMP pathway. This led to significant lactic acid accumulation, while acetic acid production via the phosphoketolase pathway remained repressed below detection limits. Untargeted metabolomics results revealed a significant downregulation of nonvolatile metabolites, including the tripeptide Ile-Ile-Tyr and *O*-acetyl-L-serine, within the 63 + 290 co-fermentation group. This reduction indicates that the microbial community efficiently converted nitrogen sources into flavor precursors. Simultaneously, the consumption of TCA cycle intermediates, such as fumarate, accelerated the overall carbon metabolic flux. Building upon this foundation, the proteolytic system of *L. paracasei* subsp. *paracasei* 63 supplied the co-fermentation system with abundant amino acid precursors. Through the cascade catalysis of transaminases and decarboxylases within the branched-chain amino acid degradation pathway of *Lc. cremoris* 290, these precursors ultimately generated 3-methylbutyraldehyde, the primary compound responsible for the characteristic malty aroma [40,41]. Concurrently, the redistribution of pyruvate flux was consistent with the potential activation of the diacetyl synthesis pathway, as evidenced by the significant increase in diacetyl content and established the rich lactic aroma profile [42]. Furthermore, acyl-CoA generated from fatty acid metabolism underwent highly efficient esterification with alcohols, thereby enriching the overarching fruity aroma of the product [43].

The superior flavor profile of the 63 + 290 co-fermentation group compared to the 63 + 26 co-fermentation group likely stems from the larger and more complex genome and metabolic network of *Lc. cremoris* 290. *Lc. cremoris* 290 exhibited a higher reaction-to-gene ratio. This elevated ratio indicates greater metabolic regulatory flexibility resulting from evolutionary adaptation to the fermentation environment. Within the mixed fermentation system, *L. paracasei* subsp. *paracasei* 63 and *Lc. cremoris* 290 established a unique metabolite supply and demand relationship characterized by asymmetric metabolic transfer. This relationship reflects a profound functional complementarity between the two strains. Specifically, compared to *Lc. lactis* subsp. *lactis* 26, *Lc. cremoris* 290 possesses several unique metabolic functional genes detailed in Supplementary Table S4. These specific genes play a decisive role in subsequent flavor metabolism and environmental adaptation pathways [44,45].

In the pyruvate and branched-chain amino acid metabolic branches, *Lc. cremoris* 290 possesses aminotransferase and decarboxylase gene clusters including *kdcA* and *spxB*. Among these, the multiple-substrate-specific α-keto acid decarboxylase encoded by *kdcA* catalyzes the conversion of pyruvate into acetaldehyde [46]. Furthermore, it drives the decarboxylation of branched-chain amino acids via the Ehrlich pathway. This dual capability provides a plausible genomic basis for the high accumulation of diacetyl and 3-methylbutyraldehyde within this co-fermentation system [47]. Regarding nitrogen metabolism and acid stress tolerance, the *Lc. cremoris* 290 genome contains strain-specific core components of the ADI pathway, namely *arcD* and *arcC*. The efficient arginine uptake mediated by *arcD* provides genomic evidence for the arginine cross-feeding observed during the metabolomics analysis [48]. Additionally, its coupled proton consumption and ATP generation mechanism significantly enhances the acid tolerance of the strain during the late fermentation phase.

While the alignment between GEM predictions and metabolomic profiles provides strong suggestive evidence for metabolic synergy, it is important to acknowledge that untargeted metabolomics captures a “snapshot” of metabolites rather than direct metabolic flux. Therefore, the proposed cross-feeding mechanisms should be interpreted as highly probable metabolic hypotheses derived from genomic potential and end-product analysis. Further studies employing ^13^C-based fluxomics or targeted gene deletions would be instrumental in providing definitive quantification of these inter-species metabolic exchanges. While this study serves as a proof-of-concept, the application of this computational framework in industrial strain selection still requires large-scale blind trials to validate its broader generalizability and to carefully evaluate the cost–benefit ratio.

These findings collectively indicate that the complex metabolic interaction network established between *L. paracasei* subsp. *paracasei* 63 and *Lc. cremoris* 290 enables efficient metabolite conversion through profound cascading effects. Consequently, this synergy comprehensively enhances the flavor profile and nutritional quality of the fermented milk. By elucidating the metabolic complementarity between the two strains, this study provides a mechanistic hypothesis for the exceptional fermentation performance of the 63 + 290 co-fermentation group. Ultimately, this research provides a preliminary theoretical framework for the rational design of starter cultures and the targeted flavor regulation of fermented dairy products. Specifically, given the significant enhancement in the production of diacetyl and 3-methylbutyraldehyde, which contribute to characteristic buttery and malty notes, this co-culture system exhibits great potential for application in the commercial production of flavored yogurts, sour cream, buttermilk, and certain cheese varieties (e.g., Cheddar or Gouda) where these specific sensory attributes are highly desired.

## 5. Conclusions

This study integrated GEMs for predictive analysis and experimentally demonstrated that, among the limited cohort of tested strains, *Lc. cremoris* 290 serves as the optimal co-fermentation strain for *L. paracasei* subsp. *paracasei* 63. Within this mixed fermentation system, these two strains established a close complementary nutritional network centered on amino acid exchange. The modulation of nitrogen, carbon, and lipid metabolism, alongside the redistribution of pyruvate metabolic flux, effectively promoted the formation of characteristic fruity, malty, and milky flavors. Although the untargeted metabolomic analysis strongly supports the predicted metabolic interactions, further studies utilizing targeted metabolite quantification or metabolic flux analysis remain necessary to conclusively confirm these underlying mechanisms. In summary, this work provides a proof-of-concept framework for the rational design of starter cultures within dairy fermentation.

## Figures and Tables

**Table 1:** List of sample numbers and their inoculated strains.

Sample Number	Inoculated Strain
63	*L. paracasei* subsp. *paracasei* 63
26	*Lc. lactis* subsp. *lactis* 26
290	*Lc. cremoris* 290
63 + 26	*L. paracasei* subsp. *paracasei* 63 + *Lc. lactis* subsp. *lactis* 26
63 + 290	*L. paracasei* subsp. *paracasei* 63 + *Lc. cremoris* 290

**Table 2:** Acidity, pH, fermentation time and viable cell counts of different fermented milk samples.

		EF				ER			
Sample ID	Curd Time ^1^/h	pH	TA/°T	Viable Bacterial Count/log (CFU/mL)		pH	TA/°T	Viable Bacterial Count/log (CFU/mL)	
				*L. paracasei* subsp. *paracasei* 63	*Lactococcus*			*L. paracasei* subsp. *paracasei* 63	*Lactococcus*
Single-strain fermentation groups									
63	25.5 ± 0.00	4.87 ± 0.04	62.46 ± 2.34	9.25 ± 0.07	-	4.83 ± 0.06	62.67 ± 2.31	9.32 ± 0.01	-
26	8.5 ± 0.00	4.45 ± 0.03	74.62 ± 1.20	- ^2^	8.94 ± 0.13	4.44 ± 0.02	76.21 ± 1.04	-	9.14 ± 0.06
290	48.0 ± 0.00	5.11 ± 0.02	51.83 ± 0.12	-	8.60 ± 0.04	5.02 ± 0.01	53.68 ± 0.11	-	8.59 ± 0.06
Co-fermentation group									
63 + 26	8.5 ± 0.00	4.47 ± 0.01	77.13 ± 2.36	8.24 ± 0.05	8.93 ± 0.06	4.38 ± 0.01	79.47 ± 0.18	8.04 ± 0.13	9.13 ± 0.01
63 + 290	13.5 ± 0.00	4.56 ± 0.03	71.67 ± 0.16	8.71 ± 0.05	9.32 ± 0.03	4.45 ± 0.02	74.47 ± 1.36	8.75 ± 0.08	9.26 ± 0.03

^1^ “Curd time” refers to the total fermentation duration required for the milk to completely coagulate, which was determined visually by the inversion method (i.e., the coagulum remains intact without falling when the container is inverted), corresponding to the EF. ^2^ “-” indicates single-strain fermentation where the target strain was not inoculated, hence no viable cell count data is available.

## Data Availability

The original contributions presented in this study are included in the article/Supplementary Material. Further inquiries can be directed to the corresponding author.

## Supplementary Materials

The following supporting information can be downloaded at: https://www.mdpi.com/article/10.3390/foods15111863/s1, Figure S1: Permutation test of the partial least squares discriminant analysis (PLS-DA) model for volatile compound profiles (*n* = 200). The y-axis intercepts of R2 (green circles) and Q2 (blue squares) are 0.537 and −0.487, respectively. A Q2 intercept less than 0 indicates that the model does not overfit and has high reliability; Figure S2: Permutation test of the partial least squares discriminant analysis (PLS-DA) model for untargeted metabolomic profiles (*n* = 200). The y-axis intercepts of R2 (green circles) and Q2 (blue squares) were 0.2595 and −0.5119, respectively. A Q2 intercept less than 0 indicates that the model is not overfitted and is highly reliable; Table S1: Sequencing data quality and genome assembly indicators of the tested strains; Table S2: Identification and relative quantification of volatile compounds detected by HS-SPME-GC-MS in different fermented milk groups; Table S3: Differential metabolites analysis between the 63 + 290 co-fermentation group and 63 group; Table S4: Unique genes of Lc. cremoris 290 relative to Lc. *lactis* subsp. *lactis* 26.
