# Supplementary material for: Genome-Scale Metabolic Models Guided Improvement of Fermented Milk Quality and Flavor by Lacticaseibacillus paracasei subsp. paracasei 63
Source: Foods. 2026 May 25;15(11):1863. doi: 10.3390/foods15111863 (PMC13257052; doi:10.3390/foods15111863)
Supplement: Supplementary file 1 [file foods-15-01863-s001.zip › Supplementary Materials 01.pdf]

3.1. Genome Sequencing and Assembly Quality Assessment

Table S1. Sequencing data quality and genome assembly indicators of the tested strains.

Category	Metrics	*L. paracasei* subsp. *paracasei 63*	*Lc. lactis* subsp. *lactis* 26	*Lc. cremoris* 290
NGS	Clean Bases (Gb)	1.17	0.99	1.07
	Clean Q30 (%)	97.12	97.55	97.55
	Estimated depth (×)	362	372	392
TGS	Total Bases (Gb)	0.39	0.30	0.35
	Read N50 (kb)	10.94	10.95	10.55
	Estimated depth (×)	120.5	114.1	129.6
Genome Assembly	Genome size (Mb)	3.23	2.65	2.72
	GC content (%)	46.18	35.08	35.69
	Number of contigs	1 chr + 1 pla	1 chr + 5 pla	1 chr + 7 pla
Quality Assessment	BUSCO completeness (%)	99.2	98.4	98.4
	CheckM completeness (%)	99.46	100	100
	CheckM contamination (%)	0	0.57	0.94

Chr, chromosome; pla, plasmid(s).

3.4. Differences in volatile compound profiles between single-strain fermented milk and co-cultured fermented milk

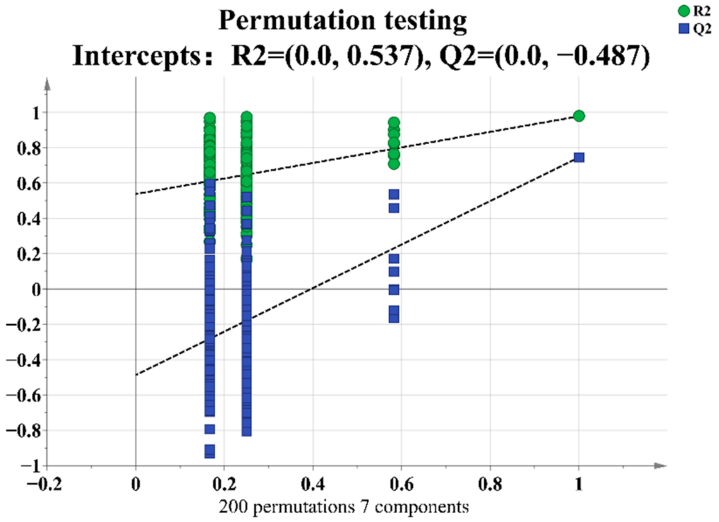

Figure S1. Permutation test of the partial least squares discriminant analysis (PLS-DA) model for volatile compound profiles (n = 200). The y-axis intercepts of R^2 (green circles) and Q^2 (blue squares) are 0.537 and -0.487, respectively. A Q^2 intercept less than 0 indicates that the model does not overfit and has high reliability.

3.5. Differences in metabolomic profiles between single-strain fermented milk and co-cultured fermented milk

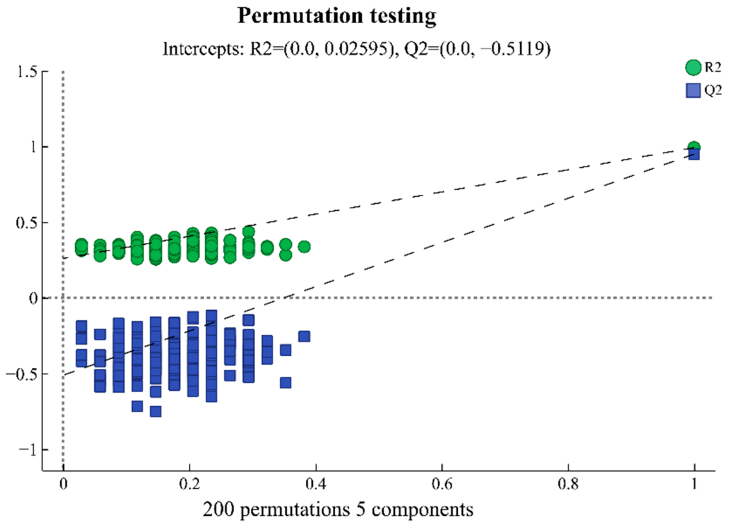

Figure S2. Permutation test of the partial least squares discriminant analysis (PLS-DA) model for untargeted metabolomic profiles (n = 200). The y-axis intercepts of R^2 (green circles) and Q^2 (blue squares) were 0.2595 and -0.5119, respectively. A Q^2 intercept less than 0 indicates that the model is not overfitted and is highly reliable.

5. Conclusions

Table S3. Differential metabolites analysis between the 63 + 290 co-fermentation group and 63 group.

Compound name	Relative abundance change
Methyl (Z)-octadec-6-enoate	↑
3-Methylbutanal	↑
Pentan-1-ol	↑
Butane-2,3-dione	↑
Methyl 16-methylheptadecanoate	↑
Methyl octadec-10-enoate	↓
2-Phenylacetaldehyde	↑
(3-Methyloxiran-2-yl)methanol	↑
2-(4-Amino-5,6-dimethylthieno[2,3-d]pyrimidin-1-ium-2-yl)sulfanyl-N-cyclohexylacetamide	↑
5-(2-Aminoethyl)-1H-imidazole-2-carbaldehyde	↓
4-Methyl-2-oxopentanoic acid	↑
Ala Pro Ala Tyr Ser	↓
Taurocholic Acid	↓

Pip(5-Iso Pgf2Vi/18:0)	↓
17-Dimethylaminogeldanamycin	↓
Coagulin R 3-Glucoside	↓
Pgp(Pgf1Alpha/22:4(7Z,10Z,13Z,16Z))	↓
Vaniprevir	↓
Pa(Pgf1Alpha/22:5(4Z,7Z,10Z,13Z,16Z))	↓
Ile-Ile-Tyr	↓
Cdp-Dg(Pge1/I-14:0)	↓
O-Acetyl-L-Serine	↓
Glycochenodeoxycholic Acid 3-Glucuronide	↓
Thr-Ala-Tyr	↓
Cytidine	↓
25-Desacetyl Rifapentine	↓
Cytosine	↓
Pulchinenoside A	↓
N-Succinyl-Leu-Leu-Val-Tyr-7-Amido-4-Methylcoumarin, >=90% (Hplc)	↓
Officinalisnin I	↓
Acetyldiphenylalanyl-Leucyl-Aspartyl-Isoleucyl-Isoleucyl-Tryptophan	↓
Pc(5-Iso Pgf2Vi/2:0)	↓
Fumaric Acid	↓
Pisumionoside	↓
7,8-Dihydrovomifoliol 9-[Apiosyl-(1->6)-Glucoside]	↓
Glycocholate Glucuronide	↓
Lucyoside M	↓
Ziziphin	↓
Amastatin	↓
Ile Val Glu	↓
Taurodehydrocholic Acid	↓
Pg(Pgf2Alpha/18:3(9Z,12Z,15Z))	↓
Pgp(Pgj2/I-15:0)	↓
N-((Hexahydro-1-Azepinyl)Carbonyl)-Leucyl(1-Methyl)-Tryptophyl-	↓

Tryptophan	
Ganoderic Acid F	↓
Asparasaponin Ii	↓
N-Acetyl-Dl-Tryptophan	↓
Chinenoside Ii	↓
Ser-Leu	↓
Trans-Beta-Farnesene	↓
Pe-Cer(14:3_2O/12:1_O)	↓
11-Beta-Hydroxyandrosterone-3-Glucuronide	↓
Val Ser Ser Ser Leu	↓
Pe(Pgf2Alpha/Dime(9,5))	↓
(4-(Beta-D-Glucopyranosyloxy)Phenyl)Acetic Acid	↓
N-L-Gamma-Glutamyl-L-Leucine	↓
Ile-Val-Ile	↓
Ethyl 2-(4-(T-Boc)Piperazin-1-Yl)-4-Hydroxypyrimidine-5-Carboxylate	↓
Magnoflorine	↓
Sanguisorbin E	↓
Ce(Pgd2)	↑
Bpd-Ma	↓
Ganoderic Acid Theta	↓
Dibekacin	↓
Isovalerylglucuronide	↓
(1Aalpha,2Beta,3Alpha,11Calpha)-1A,2,3,11C-Tetrahydro-6,11-Dimethylbenzo[6,7]Phenanthro[3,4-B]Oxirene-2,3-Diol	↓
Ceanothine B	↓
Bisindolylmaleimide I	↓
Dynorphin A (6-8)	↓
Physagulin E	↓
Oleoyl-L-Alpha-Lysophosphatidic Acid	↑
Phenylalanyl-Methionine	↓
Maleic Hydrazide	↑

[(4-Chloro-2,5-Dimethylphenyl)Thio]Acetic Acid	↑
Polypodoside C	↓
Isopentenyl Adenosine	↓
3-[(5-Nitro-1H-Indol-3-Yl)Methylidene]-1H-Indol-2-One	↓
3-[5-(2-Methylpropyl)-3,6-Dioxopiperazin-2-Yl]Propanoic Acid	↓
4-Hydroxybenzaldehyde	↑
Aerobactin	↓

↑ Indicates upregulation in the 63 + 290 co-fermentation group compared to the 63 group；

↓ Indicates downregulation in the 63 + 290 co-fermentation group compared to the 63 group.
